# The Relationship between Trial-by-Trial Variability and Oscillations of Cortical Population Activity

**DOI:** 10.1038/s41598-019-53270-7

**Published:** 2019-11-15

**Authors:** Edan Daniel, Thomas Meindertsma, Ayelet Arazi, Tobias H. Donner, Ilan Dinstein

**Affiliations:** 1 Department of brain and cognitive science, Ben Gurion University of the Negev, Beer-Sheva, Israel; 2 Department of psychology, Ben Gurion University of the Negev, Beer-Sheva, Israel; 3 Zlotowski center for neuroscience, Ben Gurion University of the Negev, Beer-Sheva, Israel; 4 Department of Neurophysiology and Pathophysiology, University Medical Center Hamburg-Eppendorf, Hamburg, Germany; 5 Department of Psychology, University of Amsterdam, Amsterdam, The Netherlands; 6 Amsterdam Brain and Cognition (ABC), University of Amsterdam, Amsterdam, The Netherlands

**Keywords:** Perception, Cognitive neuroscience, Sensory processing, Visual system

## Abstract

Neural activity fluctuates over time, creating considerable variability across trials. This trial-by-trial neural variability is dramatically reduced (“quenched”) after the presentation of sensory stimuli. Likewise, the power of neural oscillations, primarily in the alpha-beta band, is also reduced after stimulus onset. Despite their similarity, these phenomena have so far been studied and discussed independently. We hypothesized that the two phenomena are tightly coupled in electrophysiological recordings of large cortical neural populations. To test this, we examined magnetoencephalography (MEG) recordings of healthy subjects viewing repeated presentations of a visual stimulus. The timing, amplitude, and spatial topography of variability-quenching and power-suppression were remarkably similar. Neural variability quenching was eliminated by excluding the alpha-beta band from the recordings, but not by excluding other frequency-bands. Moreover, individual magnitudes of alpha-beta band-power explained 86% of between-subject differences in variability quenching. An alternative mechanism that may generate variability quenching is increased phase alignment across trials. However, changes in inter-trial-phase-coherence (ITPC) exhibited distinct timing and no correlations with the magnitude of variability quenching in individual participants. These results reveal that neural variability quenching is tightly coupled with stimulus-induced changes in the power of alpha-beta band oscillations, associating two phenomena that have so far been studied in isolation.

## Introduction

Neural activity is highly variable, such that repeated presentations of an identical stimulus result in variable neural responses across trials^1–6^. This trial-by-trial variability is relatively large before stimulus presentation, and strongly reduced (quenched) approximately 200 ms after stimulus presentation^7^. Neural variability quenching is a robust phenomenon that has been reported in intracellular membrane potential recordings in cats, extracellular recordings of spiking activity in monkeys^7,8^, and in human electroencephalography (EEG)^9–12^, electrocorticography (ECOG)^13^, magnetoencephalography (MEG)^11^, and functional magnetic resonance imaging (fMRI) recordings^14,15^. Furthermore, the phenomenon was reported during both awake and anaesthetized states, and in several cortical areas^7,15^ using a variety of sensory stimuli^7,10^. Neural variability quenching seems to be a network phenomenon that is apparent across large populations of neighboring neurons regardless of their firing rates or stimulus selectivity^7,16^.

Another robust phenomenon that is apparent in recordings of electrophysiological mass activity is the reduction of induced oscillatory power approximately 200 ms after stimulus presentation. This power suppression predominates in the alpha (8–13 Hz) and beta (14–25 Hz) bands, and is often referred to as event related desynchronization (ERD)^17,18^. It is evident in a spatially selective manner corresponding to the sensory-activated cortical areas^19^, and coincides with increases in gamma power (>30 Hz) and population spiking activity^20^. It is, therefore, commonly assumed that reductions in alpha power indicate an increase in cortical activity^21^.

**Figure 1:**
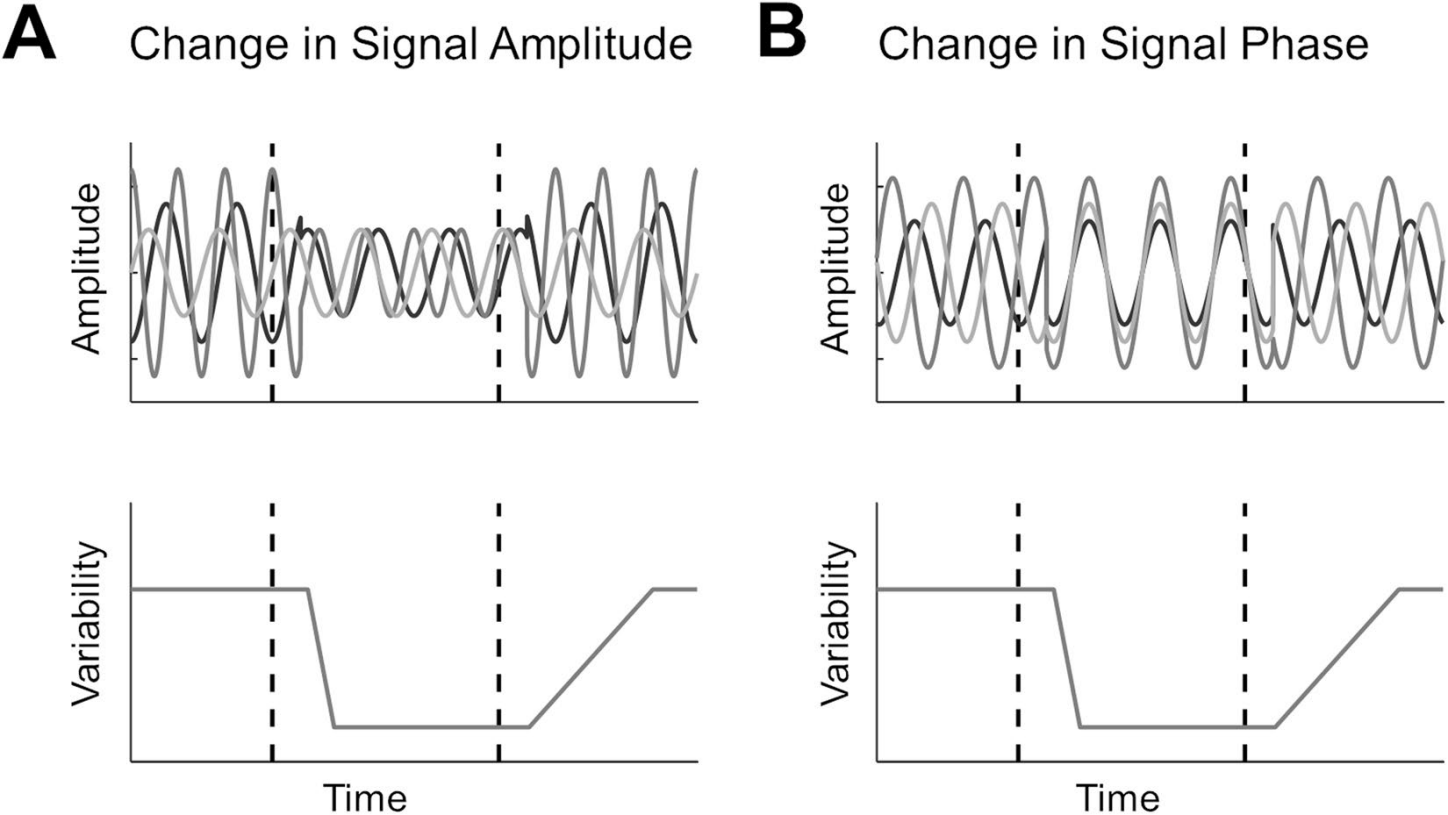
Schematic illustration of two different mechanisms for reducing trial-by-trial variability. Hypothetical oscillatory activity in three independent trials is presented with respect to stimulus presentation (top panels). Dashed lines mark times of stimulus onset and offset, respectively. Reducing the amplitude of oscillations (A, top panel) or aligning their phase (B, top panel) will create a reduction in trial-by-trial neural variability (bottom panels). Note that the two options are not mutually exclusive and affect variability in an indepedent manner.

In the current study we examined neural variability quenching in the context of mass electrophysiological recordings using MEG. In such recordings, trial-by-trial variability may be driven by two independent mechanisms (Fig. 1). First, a stimulus-induced decrease in oscillatory power/amplitude would yield fewer trail-by-trial differences regardless of the precise timing of these oscillations (Fig. 1A). Second, a stimulus-evoked increase in phase coherence across trials (i.e., better phase locking across trials) would also yield fewer trial-by-trial differences (Fig. 1B). The two mechanisms are not mutually exclusive and may both contribute to the variability quenching phenomenon as recorded in EEG or MEG studies^22^. Note that the two mechanisms may also counteract each other such that, for example, variability may increase despite a partial increase in phase coherence, if it is accompanied by a simultaneous large increase in oscillatory power.

In the current study we quantified the relationships between spectral power and trial-by-trial neural variability in several ways. First, we extracted specific frequency bands from the MEG data and determined the effect that this had on neural variability magnitudes. Second, we examined whether individual subject differences in spectral power could explain individual differences in neural variability. Finally, we examined whether individual subject differences in inter-trial phase coherence (ITPC) could explain individual differences in neural variability. These analyses were performed using MEG recordings from an experiment with a coherently moving stimulus (henceforth called ‘moving pattern’) presented for a relatively long duration (750 ms), which is known to induce reliable sustained gamma band responses in MEG recordings^23^. Such gamma band responses are difficult to detect with other techniques or stimuli^24,25^.

## Materials and Methods

The current study utilized a subset of MEG recordings that were part of a previously published study regarding perceptual decision making^23^.

### Subjects

23 subjects (13 females; age range, 20–54; mean age, 26.6 years; SD, 7.5 years) were included in the current study. All subjects had normal or corrected-to-normal vision and no known history of neurological disorders. The experiment was conducted in accordance with the Declaration of Helsinki and approved by the local ethics committee of the Hamburg Medical Association. Each subject gave written informed consent.

### Experimental design

**Figure 2:**
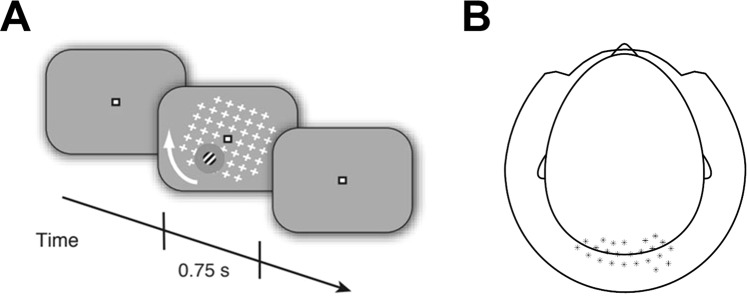
Experimental design. (**A)** Illustration of the stimulus presented to the subjects. A moving pattern of white crosses was presented along with a peripheral Gabor patch located in either the left of right bottom quadrants of the visual field. Each trial began with a fixation mark for 250 ms, followed by a fixation mark and the stimulus for 750 ms, the fixation mark only for 750 ms, and finally an inter-trial interval with a blank screen for 750 ms. (**B)** Scalp map indicating the location of chosen sensors that were used in subsequent analyses.

Subjects passively viewed a repeating visual stimulus while MEG data was recorded (Fig. 2A). The stimulus consisted of a large and salient, full-field grid of white crosses (17° × 17°) that rotated in the clock-wise or counter-clockwise direction (speed: 160°/s). This moving pattern surrounded a small Gabor patch (diameter, 2°; two cycles), located in the lower right or left visual field quadrant (counterbalanced between subjects) that was modulated at a temporal frequency of 10 Hz. Subjects fixated on a fixation mark (0.8° width and length) in the middle of the screen to suppress potential eye movements. Stimuli were presented using the Presentation Software (NeuroBehavioral Systems Inc.). Stimuli were back-projected on a transparent screen using a Sanyo PLC-XP51 projector with a resolution of 1024 × 768 pixels at 60 Hz. Subjects were seated 58 cm from the screen in a whole-head magnetoencephalography (MEG) scanner setup in a dimly lit room. Each trial started with the presentation of the fixation mark for 250 ms, followed by fixation mark and full stimulus for 750 ms, fixation mark only for 750 ms, and an inter-trial-interval of 750 ms containing a blank screen. This experiment was used as a localizer for quantifying sensory responses to the moving pattern and Gabor patch in a previous study^23^. This previous study examined perceptual decision making during motion induced blindness^26^, a phenomenon where the moving pattern induces the illusory disappearances of small but salient static stimuli (i.e., the Gabor patch). The flicker of the Gabor patch in this localizer, however, was specifically implemented to prevent the occurrence of motion induced blindness^23^. The large sensory cortical response induced by this compound stimulus was mostly due to the larger moving pattern rather than the small Gabor patch^27^.

### Data acquisition

MEG data were acquired using a 275-channel MEG system (VSM/CTF Systems) with a sample rate of 1200 Hz, while subjects were in a seated position. The location of each subjects’ head was measured throughout the experiment using three fiducial markers placed on both ears and the nasal bridge to control for excessive movement. Furthermore, electrooculography and electrocardiography were recorded to aid post-hoc artifact rejection.

### Preprocessing

MEG data were analyzed in MATLAB (MathWorks Inc., USA) using the Fieldtrip toolbox^28^, EEG toolbox^29^, and custom-written software. Each trial was defined as an epoch that started 800 ms before stimulus onset and lasted until 1000 ms after stimulus offset (i.e., −800 to 1750 ms with respect to stimulus onset). Artifacts related to environmental noise, eye and muscle activity, and squid jumps were detected using standard automated artifact rejection methods included in the Fieldtrip toolbox. Trials containing artifacts were excluded and remaining data was down sampled to 500 Hz. The final analysis was conducted, on average, with 156 trials (SD = 54.6) per subject. We focused our analysis on the cortical regions processing the physical stimulus (i.e., visual cortex). We, therefore, selected 25 occipital and parietal sensors that exhibited the strongest stimulus-induced response, as defined and previously reported by Meindertsma *et al*. (2017) (Fig. 2B). One sensor was missing in many subjects, and therefore removed from the data of all subjects, resulting in 24 sensors of interest. We also present topographical displays of our findings, which demonstrate the spatial selectivity of the results.

### Spectral analyses

Spectral decomposition of MEG recordings was performed using a sliding Hamming windowed Fourier transform (step size: 20 samples, window length: 250 samples, centered in the middle), as implemented in EEGLAB^29^, and performed separately for each trial, sensor, and subject. This window size enabled us to estimate power in the delta-band (2–4 Hz), while retaining a reasonable temporal resolution for assessing stimulus-induced changes in power. The same window properties were used across all frequency bands to allow comparison of their temporal dynamics.

Power was calculated for each time-frequency segment by computing the absolute values of the Fourier coefficients. The resulting time–frequency power estimates (i.e., spectrograms) were averaged across the 24 sensors described above and then across trials to obtain a spectrogram for each subject. These were then averaged to create a spectrogram for the entire group. We also isolated power changes in specific frequency bands, which included the delta (2–4 Hz), alpha-beta (5–25 Hz), and gamma (60–120 Hz) bands, using band-pass filters. This was implemented by applying one low-pass and one high-pass Hamming-windowed FIR filter for each of the frequency ranges, with cutoff frequencies equal to the upper and lower limits of each frequency range, respectively. Topographic plots of changes in power were obtained by computing the mean power of each frequency band 200–700 ms after stimulus onset, relative to baseline (−250 ms to stimulus onset), in units of percent signal change (see below). These power changes were then averaged across subjects to create a single topographic plot.

To estimate the effect of extracting each of the frequency bands on trial-by-trial variability we used band-stop finite impulse response (FIR) filters with a Hamming-window. The cutoff frequencies of each filter were equal to the lower and upper limits of each frequency range described above.

Relative stimulus-induced change in power was normalized into units of percent signal change. We calculated the power in the pre-stimulus $(Powe{r}_{pre})$ period (−250 ms to stimulus onset) and post-stimulus $(Powe{r}_{post})$ period (200–700 ms after stimulus onset) and computed percent signal change as follows:

$$Relative\,change\,in\,power=(\frac{Powe{r}_{post}}{Powe{r}_{pre}}-1)\cdot 100$$

Finally, we also estimated inter-trial phase coherence (ITPC) across trials, for each frequency and sensor^29^. This measure reflects the degree to which the phase of each frequency is aligned across trials. The ITPC measure was calculated as follows:

$$ITPC\,(f,t)=\frac{1}{n}\mathop{\sum }\limits_{k=1}^{n}\frac{{F}_{k}(f,t)}{|{F}_{k}(f,t)|}$$

Raw ITPC was calculated for each frequency (f) in a sliding time window (t) that was equivalent to that described above for the frequency analysis. F stands for the Fourier transform, k is the trial number, and | | is the complex norm. The raw ITPC values in each time window can range from 0 (indicating complete lack of phase alignment across trials) to 1 (indicating perfect phase alignment). ITPC values were then normalized to percent change units with respect to the pre-stimulus period as described above for the power calculations.

### Analyses of trial-by-trial variability

Trial-by-trial variability was computed across trials for each time point in every sensor. Absolute trial-by-trial variability in the pre-stimulus ($Va{r}_{pre}$) and post-stimulus ($Va{r}_{post}$) periods were computed by averaging across the relevant time-points (−250 ms to stimulus onset, and 200–700 ms after stimulus onset, respectively). Relative change in trial-by-trial variability (i.e., neural variability quenching) was then estimated by dividing the variability in the post-stimulus period by the pre-stimulus period and adjusting to percentage change units, as follows:

$$Neural\,Variability\,Quenching=(\frac{Va{r}_{post}}{Va{r}_{pre}}-1)\cdot \,100$$

To isolate the contribution of each frequency band to the magnitude of variability quenching, we used Hamming windowed FIR band-stop filters to exclude data in each of the frequency bands as described above.

### Head motion control analysis

To control for potential head motion artifacts, we computed the three-dimensional position of the head in every time-point. We then quantified head-motion by computing the mean absolute difference in position from each time-point to the next. Estimated magnitudes of head-motion were then correlated with individual measures of neural variability quenching to determine potential relationships or lack there-of.

### Statistical analysis

To identify statistically significant changes in oscillatory power or trial-by-trial variability, while correcting for multiple comparisons, we used two-tailed cluster-based permutation tests^30^. This involved identifying time-points with an un-corrected p-value smaller than 0.05 when applying a paired sample t-test. Consecutive time-points that exceeded the threshold formed candidate clusters and the sum of each cluster’s t-values was computed. We then used a Monte-Carlo permutation with 1000 iterations to define a probability distribution of t-value sums from clusters in randomly drawn sets of time-points^30^. The corrected p-value was defined by the relative percentile of the actual cluster statistic relative to this null distribution of random cluster statistics.

We computed Pearson’s correlation coefficients to assess potential relationships between neural variability quenching and oscillatory power or ITPC, across subjects. The same analysis was performed in the control analysis with estimates of head motion. We also used a partial-correlation analysis to estimate the relative contribution of oscillatory power in each frequency band to the magnitude of neural variability. This eliminated inter-dependencies across frequency bands, thereby isolating the contribution of each frequency band from that of the others. We performed a regression analysis to explain between-subject-differences in the magnitude of variability quenching, using the subjects’ change in power as measured in each frequency band separately. We then computed penalized-likelihood criteria for comparison between the regression models using both the Akaike Information Criterion (AIC) and Bayesian Information Criterion (BIC) as implemented in the MATLAB *fitlm* function. Spatial correlations between changes in power and variability quenching were calculated for each subject, and then averaged across subjects.

## Results

**Figure 3:**
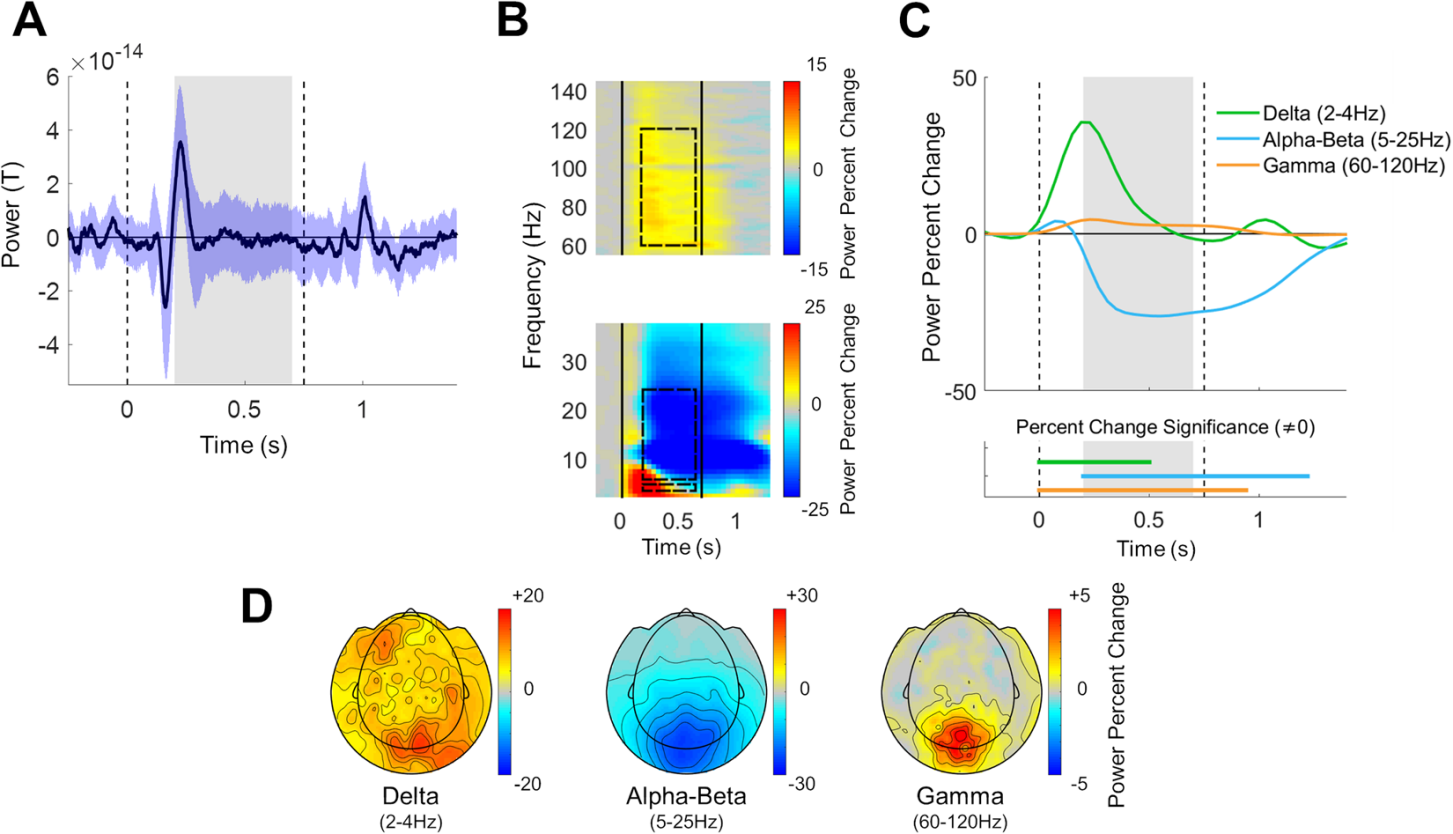
Stimulus-evoked Event Related Field (ERF) and stimulus-induced spectral responses in the different frequency bands. **(A)** Grand average ERF across trials and subjects (black line) demonstrating the stimulus evoked response. Blue shaded area presents the standard deviation across subjects. (**B)** Spectrogram demonstrating the relative change in oscillatory power with respect to the pre-stimulus period (−250 ms to stimulus onset). Black vertical lines: stimulus onset and offset. Dashed rectangles: selected frequency bands and time window. (**C)** Temporal changes in the power of each frequency band, averaged across the selected sensors, all trials, and subjects. Dashed vertical lines: stimulus onset and offset. Gray filling: window over which neural variability quenching was computed in subsequent analyses. Horizontal lines on bottom indicate time segments where the change in power of each band was significantly different from zero (p < 0.05, two-tailed permutation test, cluster corrected). (**D)** Topographic maps of mean power change 200–700 ms after stimulus presentation, relative to the pre-stimulus period in units of percent signal change (averaged across trials and subjects).

Subjects exhibited clear stimulus-evoked ERF responses (Fig. 3A) with commonly reported negative and positive peaks that were locked to stimulus onset and offset. In addition, subjects exhibited strong stimulus-induced responses (Fig. 3B) with a characteristic time-frequency and spatial signature^31^. An initial broadband power increase in all frequencies was followed by different frequency-band specific dynamics. Power in the delta (2–4 Hz) band increased dramatically with stimulus presentation, peaking approximately 200 ms after stimulus onset. Power in the alpha-beta frequency band (5–25 Hz) increased transiently and then decreased to negative values ~200 ms after stimulus presentation (Fig. 3C). Power in the gamma (60–120 Hz) band increased after stimulus presentation in a sustained manner and returned to pre-stimulus levels ~250 ms after stimulus offset.

Note the similar temporal dynamics of power across the alpha-beta (5–25 Hz) frequency range (Fig. 3B). This motivated us to simplify all further analyses by aggregating this frequency range together, as performed in other recent studies^32,33^. Importantly, analyzing the alpha and beta bands separately did not change any of the reported conclusions. Power changes in the three selected frequency bands, 200–700 ms after stimulus presentation, exhibited different spatial characteristics (Fig. 3D). Power reduction in the alpha-beta band and power enhancement in the gamma band were specific to sensors located over occipital and parietal cortices, while changes in the delta band were more diffused and distributed.

### Neural variability quenching

**Figure 4:**
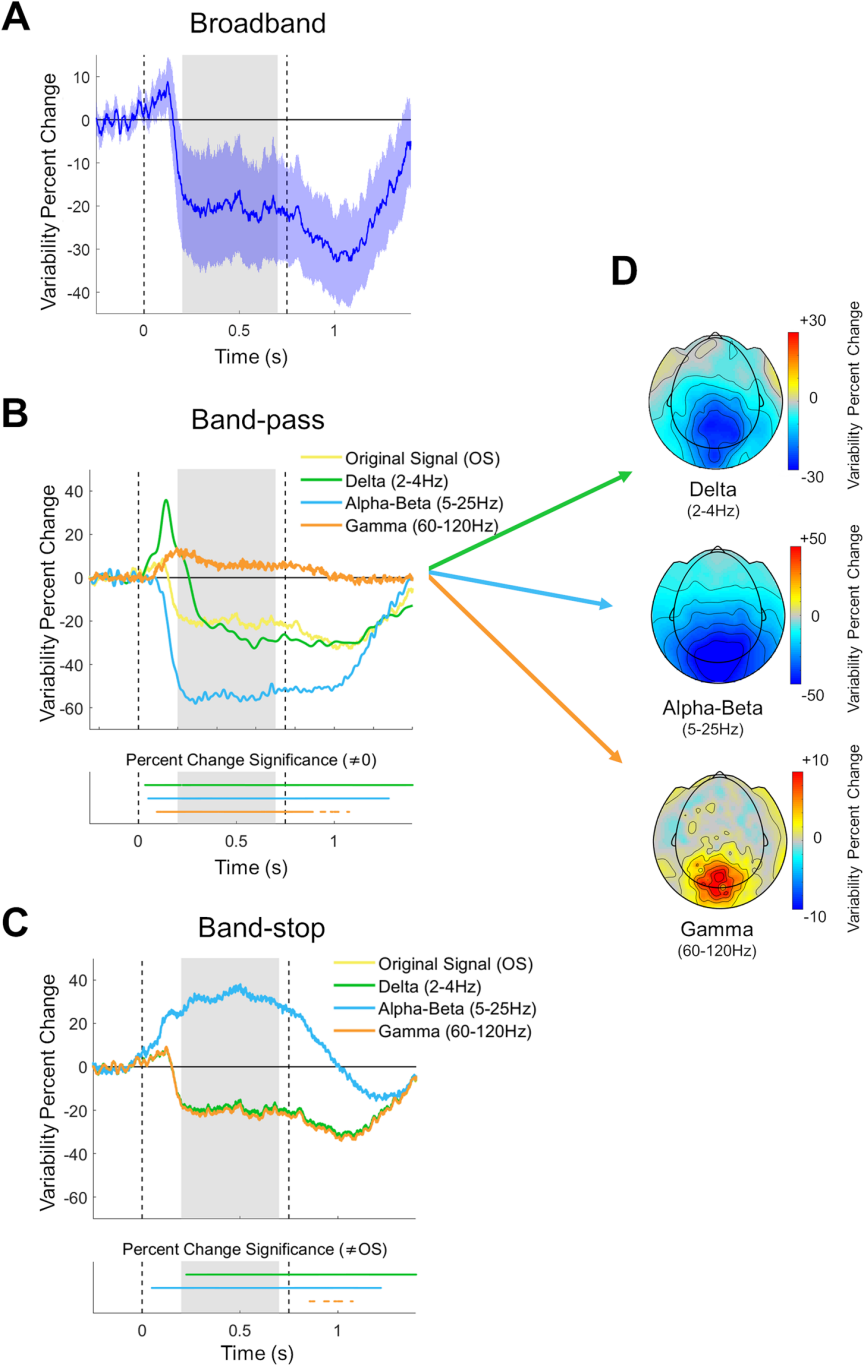
Trial-by-trial neural variability changes following stimulus presentation. **(A)** Neural variability over time in units of percent change relative to the pre-stimulus period, averaged across selected sensors and all subjects. Blue shaded area: confidence interval across subjects. Dashed vertical lines: stimulus onset and offset. Gray background: window with sustained neural variability quenching. **(B**) Neural variability quenching within each isolated frequency band (delta, alpha-beta, or gamma) when using band-pass filters. Time segments where variability was significantly different from zero are marked in the lower panel (p < 0.05, two-tailed permutation test, cluster corrected). (**C)** Neural variability quenching after eliminating each frequency band from the data using band-stop filters. Time segments where variability was significantly different from that in the original signal (OS) are marked in the lower panel (p < 0.05, two-tailed permutation test, cluster corrected). (**D**) Topographic maps of neural variability changes 200–700 ms after stimulus presentation, relative to the pre-stimulus period, after isolating each of the frequency bands.

Subjects exhibited robust reductions in trial-by-trial neural variability 200–700 ms after stimulus presentation in comparison to the pre-stimulus period (Fig. 4A). To determine the relationship between variability quenching and the activity of specific frequency bands, we re-computed neural variability after isolating each frequency-band using band-pass filters (Fig. 4B). This revealed that variability quenching was remarkably strong 200–700 ms after stimulus presentation in isolated alpha-beta band activity, where variability quenching reached a mean value of −54.42% (blue line, Fig. 4B). In contrast, neural variability was enhanced, rather than quenched in isolated gamma band activity (+6.95%, orange line, Fig. 4B). In isolated delta band activity, there was an initial transient increase in variability, followed by a reduction in variability from approximately 150 ms after stimulus onset, reaching a mean value of −20.32% (green line, Fig. 4B). This was similar to the magnitude of variability quenching in the original, un-filtered data (−20.29%, yellow line, Fig. 4B).

In a complementary analysis we used band-stop filters to eliminate specific frequency bands in the MEG data (see Materials and Methods) and then re-computed trial-to-trial variability (Fig. 4C). Eliminating the alpha-beta frequency band dramatically altered variability quenching from a mean value of −20.29% in the original signal (yellow line, Fig. 4C) to variability enhancement with a mean value of +31.67% (blue line, Fig. 4C). In contrast, eliminating the Delta or Gamma bands had minor effects on variability quenching, which had values of −19.26% and −20.83% (green and orange lines, Fig. 4C) respectively. Taken together, these results demonstrate that variability quenching is strongly dependent on neural activity changes that are specific to the alpha-beta band. Note that this analysis estimated the mean response across subjects and disregarded individual differences.

The spatial topography of changes in neural variability was selective to occipital and parietal sensors in isolated delta, alpha-beta, and gamma band activity. The band-specific spatial changes in neural variability (Fig. 4D) were very similar to the spatial changes in power (Fig. 3D). This was apparent in a moderate spatial correlation in the delta (r(268) = 0.57, p = 0.02) band and very strong correlations in the alpha-beta (r(268) = 0.95, p < 0.001) and gamma (r(268) = 0.95, p < 0.001) bands.

### Individual differences

**Figure 5:**
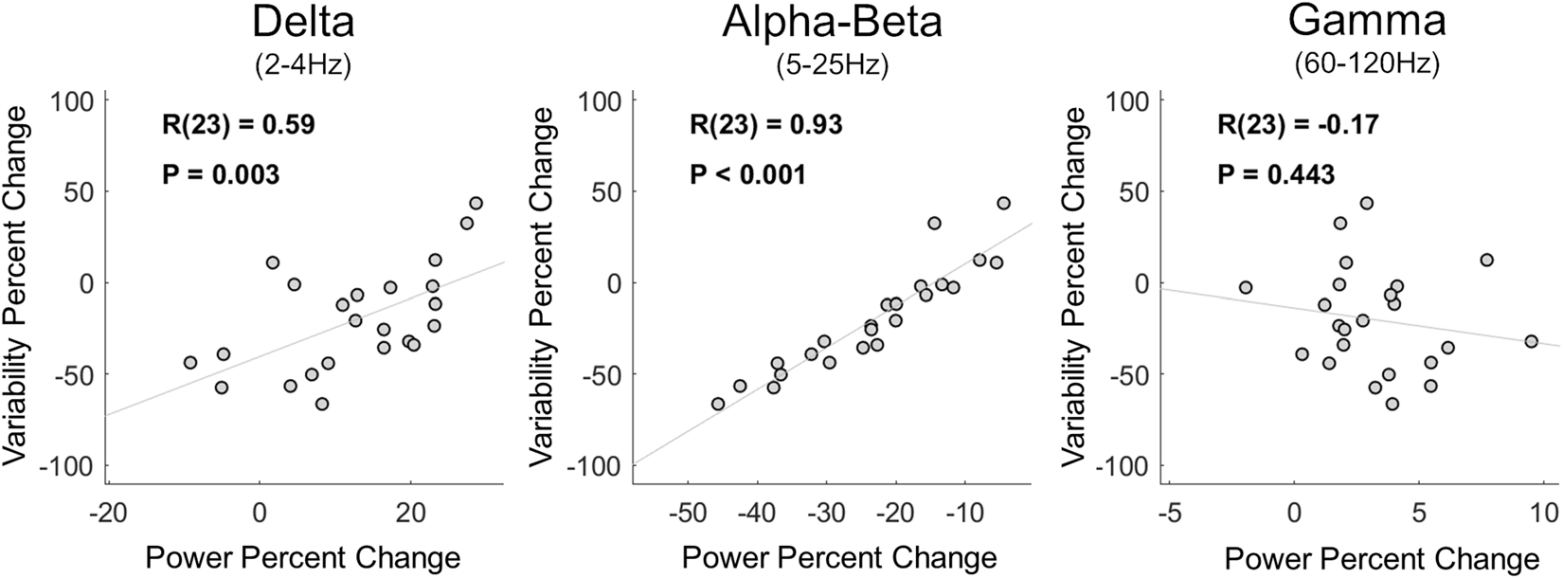
Individual magnitudes of variability-quenching were strongly correlated with relative changes in MEG power of specific frequency bands. Scatter plots demonstrating the correlation between individual magnitudes of variability quenching and changes in delta, alpha-beta, or gamma band power. Linear fit lines, Pearson’s correlation coefficients (R) and significance level (P) are presented in each panel.

In line with previous studies^10^, individual subjects exhibited distinct magnitudes of neural variability quenching. These individual differences were strongly correlated with the magnitudes of stimulus-induced power changes (Fig. 5) in the delta (r(23) = 0.59, p = 0.003) and alpha-beta (r(23) = 0.93, p < 0.001) frequency-bands, but not in the gamma band (r(23) = −0.17, p = 0.44). These results demonstrate that individual differences in variability quenching were best explained by individual differences in alpha-beta band power reductions, which explained 86% of between-subject differences (adjusted R^2^ = 0.86, AIC = 176.3, BIC = 180.9). To examine the combined predictive value of power changes in all three frequency bands on variability quenching magnitudes, we also performed a multiple regression analysis. The regression model included three predictors containing the individual subjects’ power changes in each frequency band. This regression model yielded an adjusted R^2^ value of 0.87 (AIC = 176.5, BIC = 178.8), suggesting that adding the delta and gamma power-changes did very little to improve the ability to predict individual variability quenching magnitudes.

### Variability quenching is not associated with the timing of neural responses

**Figure 6:**
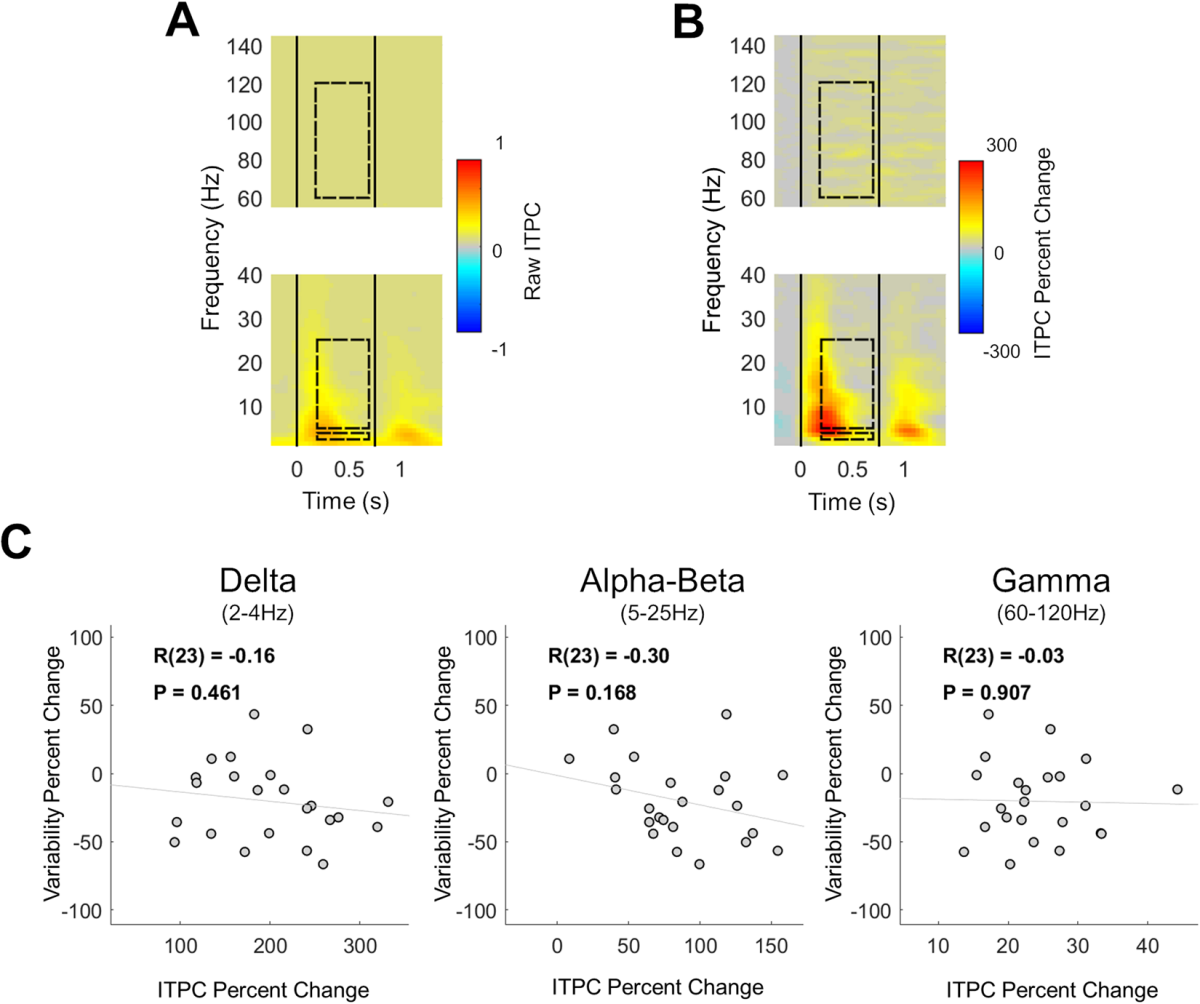
Inter-trial phase coherence (ITPC) was not associated with neural variability quenching. Time/frequency representations of stimulus-evoked changes in raw ITPC (**A**), or ITPC changes relative to the pre-stimulus baseline (**B**), revealed transient changes following stimulus onset and offset (vertical black lines), rather than sustained changes. Dashed rectangles: selected frequency bands and time window (for comparison with the previous analyses). (**C)** Scatter plots demonstrating the lack of correlations between changes in band specific ITPC and changes in neural variability, for the delta, alpha-beta, or gamma frequency bands. Linear fit lines, Pearson’s correlation coefficients (R) and significance level (P) are presented in each panel.

The results presented thus far establish that stimulus-induced modulations in alpha-beta band oscillatory power contribute to neural variability quenching. Trial-to-trial variability, however, is governed not only by the amplitude of neural oscillations (Fig. 1A), but also by their phase (i.e., timing) relative to stimulus presentation (Fig. 1B). We, therefore, tested whether variability quenching was also associated with an increase in inter trial phase coherence (ITPC). ITPC increased transiently after stimulus onset and offset (Fig. 6A,B) with a time course that corresponded to the appearance of the ERF peaks (Fig. 3A). This ITPC time course was distinct from that of variability quenching, which was sustained throughout the stimulus presentation (Fig. 4). Furthermore, individual magnitudes of variability quenching were not significantly correlated with ITPC changes in any of the examined frequency bands (Fig. 6C): delta (r(23) = −0.16, p = 0.46), alpha-beta (r(23) = −0.30, p = 0.17), or gamma (r(23) = −0.03, p = 0.91). Taken together, these results demonstrate that variability quenching is not associated with stimulus-evoked changes in the phase of neural oscillations.

### Control analyses

To exclude alternative explanations of the data we examined whether head motion affected our measures of neural variability. We computed a mean measure of head motion magnitude for each subject (see Methods) and found that there was no significant correlation with neural variability quenching (r(23) = 0.12, p = 0.54). Between-subject differences in variability quenching were, therefore, unrelated to individual differences in head movements.

## Discussion

Our results demonstrate that neural variability quenching is tightly coupled to reductions in alpha-beta band power, as measured by MEG. The timing, amplitude, and spatial topography of variability quenching and decreases in alpha-beta band power were remarkably similar (Figs 3, 4), and removing the alpha-beta band from the data entirely eliminated the variability quenching phenomenon (Fig. 4C). The strong relationship between changes in oscillatory power in this frequency band and neural variability were also apparent when examining individual subject differences (Fig. 5). Specifically, individual magnitudes of alpha-beta band power changes explained 86% of between-subject differences in neural variability quenching. In contrast, removing the delta or gamma band from the data had little effect on the magnitude of variability quenching (Fig. 4), and adding individual differences in delta or gamma band power as predictors in a multiple regression model did not increase the explanatory power beyond what was already described with alpha-beta band changes (adjusted R^2^ = 0.87). Taken together, these findings suggest that neural variability quenching is tightly coupled with changes in alpha-beta band power that are induced by the presentation of a stimulus.

While neural variability quenching was strongly related to the amplitude of induced oscillatory power, it was not related to the phase-locking of neural oscillations. The time course of ITPC changes did not correspond to the time course of neural variability quenching, and individual subject ITPC magnitudes were not correlated with neural variability quenching magnitudes (Fig. 6). Hence, cortical responses to sensory stimuli are characterized by relatively high reproducibility (i.e., low trial-by-trial variability) that is dependent on stimulus-induced decreases in alpha-beta band power rather than stimulus-evoked phase resetting.

### Event related desynchronization and synchronization

Stimulus or task induced reductions in oscillatory power, primarily in the alpha-beta band, are commonly referred to as ERD^17,18^, which is thought to coincide with increased synchronization in gamma band oscillations and increased multi-unit activity^20^. These concomitant changes in cortical oscillatory power are thought to represent the transition of cortical state from an idle “resting-state” to an active state of anticipation, sensory processing, and/or task initiation. It is believed that such oscillatory changes are essential for synchronizing the activity of task-related cortical neural ensembles^34,35^. Previous studies have not examined how these changes in oscillatory power relate to measures of trial-by-trial neural variability.

We selected the current experiment, utilizing MEG recordings and an experimental design with a relatively long, salient, moving stimulus, because it is particularly useful for identifying sustained gamma band responses^23^ that are difficult to identify with other techniques and experimental designs^24,25^. Indeed, our results revealed clear sustained gamma band responses with focal topography in occipital and parietal sensors (Fig. 3).

We initially hypothesized that we would find a positive correlation between the magnitude of ERD and neural variability quenching as well as a negative correlation between the magnitude of gamma band power and neural variability quenching. This was expected given the inverse relationship between stimulus-induced ERD and gamma power. However, we did not find a significant relationship between gamma power and neural variability quenching (Fig. 5). This suggests that individual differences in neural variability, in MEG recordings, are mostly associated with differences in alpha-beta band oscillations, which are larger in amplitude than gamma band oscillations. Note that gamma band responses have a substantially higher signal to noise ratio in invasive recordings of cortical mass activity^33^. Studying the relationship between neural oscillations and variability quenching using intra-cranial recordings is, therefore, highly warranted.

### Ongoing neural activity

Ongoing neural activity continuously changes and fluctuates in the absence of stimuli or tasks thereby creating considerable moment-by-moment neural variability^4,36,37^. Some studies have suggested that these ongoing fluctuations persist during the processing of stimuli^4^ and execution of tasks^38,39^ such that stimulus evoked responses are linearly superimposed on ongoing fluctuations. In such a case one would expect similar trial-by-trial neural variability to exist before and after stimulus presentation. Many recent studies, however, have shown that trial-by-trial neural variability is dramatically reduced following stimulus presentation^7,9–11,14–16^. This suggests that ongoing neural fluctuations do not persist, but are instead altered by the presentation of a stimulus such that trial-by-trial variability is reduced/quenched.

In mass electrophysiological recordings such as MEG, trial-by-trial variability may be governed by two distinct factors (Fig. 1). The first is the overall power of ongoing neural oscillations with random phase from trial to trial: the larger the power in each of the trials, the larger the variance across trials (Fig. 1A). The second is the phase coherence across trials: the larger the coherence, the smaller the variance across trials. Note that these two factors are not mutually exclusive (Fig. 1B)^22^.

Our results suggest that the presentation of a visual stimulus quenches trial-by-trial neural variability by reducing induced oscillatory power in the alpha-beta band, rather than by evoking a reproducible phase locked response (Figs 3–6). The strong relationship between the ERD and the variability quenching phenomena suggests that ongoing neural activity fluctuations are actively suppressed after the presentation of a sensory stimulus, perhaps to achieve a more reproducible and stable cortical state during sensory processing^11^.

### Behavioral significance

There are several similarities in the behavioral significance that has been assigned to the ERD and neural variability phenomena. For example, some have reported that allocating attention reduces neural variability across trials^14,40–42^ while others have reported that allocating attention creates ERD^43–45^. Similarly, some have reported that threshold-level stimuli are accurately perceived on trials with reduced neural variability^11,46^ while others have reported the same on trials with larger ERD^47^. Finally, individuals with lower contrast discrimination thresholds exhibited larger magnitudes of neural variability quenching, which coincided with larger ERD magnitudes^9^. All studies, except the last one, have reported one measure or the other and none have directly compared ERD and neural variability measures. We speculate that these independent studies may be reporting strongly correlated measures that seem to describe two tightly coupled phenomena.

### Limitations

Quenching of trial-by-trial neural variability has been studied with a variety of techniques including intra-cellular recordings of membrane potentials, extracellular recordings of single-unit spiking activity in animals^7^, and extra-cranial recordings in humans^9,10^. While all of these studies have shown similar variability quenching approximately 200 ms after stimulus presentation, the specific mechanisms that influence trial-by-trial variability in the spiking of single neurons may differ from those that influence trial-by-trial variability of large neural populations as measured with MEG. With that said, one recent study reported that reductions in trial-by-trial spike count variability (i.e., Fano factor) are significantly correlated with reductions in beta-band power of the local field potential, in macaque motor cortex during movement execution^48^. Further studies are necessary to bridge the gaps between different manifestations of neural variability as they appear in single cell spiking activity, local field potentials, EEG/MEG, and fMRI.

Another import limitation of the current study is the specific experimental setup and stimulus that was used. It may be the case that when employing other tasks or stimuli, the relationship between variability quenching and alpha-beta band power may change to some degree. For example, stimulating subjects with steady-state visual or auditory stimuli may alter the relative power of specific frequency bands and change the relative contribution of specific frequency bands to variability quenching^49^. In addition, changes in delta or gamma power may have a more pronounced impact on variability quenching in other experimental contexts.

## Conclusions

Our results demonstrate that stimulus-induced reductions in alpha-beta band power are tightly coupled with neural variability quenching as apparent in MEG recordings. The suppression of these oscillations, and subsequent reduction in trial-by-trial variability, may enable sensory cortices to generate more stable and reproducible neural representations of a stimulus across trials, which is likely to be beneficial for accurate perception. This appealing conceptual framework, whereby cortical responses involve an active reduction of neural “noise” (i.e., neural activity that is not related to the stimulus), is in line with signal detection theory principles^50^, and fits well with the existing literature regarding neural variability quenching and ERD. Quantifying oscillatory power, inter-trial phase coherence, and trial-by-trial variability in the same experiments will enable future studies to assess the robustness and validity of this conceptual framework across different stimuli, tasks, and recording techniques, and determine its behavioral significance.

## Data Availability

The MEG recordings utilized in the current study along with an explanation regarding their structure will be uploaded to an open access server and will be available to anyone who would like to re-analyze them.
